# Mental Health Patients’ Expectations about the Non-Medical Care They Receive in Primary Care: A Cross-Sectional Descriptive Study

**DOI:** 10.3390/healthcare8030235

**Published:** 2020-07-27

**Authors:** Valle Coronado-Vázquez, Dolores Museros-Sos, Bárbara Oliván-Blázquez, Rosa Magallón-Botaya, Juan Gómez-Salgado, María Antonia Sánchez-Calavera, Bárbara Masluk, María Josefa Gil-de-Gómez, Eva Rodríguez-Eguizábal

**Affiliations:** 1 Instituto Aragonés de Ciencias de la Salud (IACS), 50009 Zaragoza, Spain; mvcoronado@msn.com (V.C.-V.); lolita.museros@gmail.com (D.M.-S.); bmasluk@unizar.es (B.M.); jggomez@riojasalud.es (M.J.G.-d.-G.); ereguizabal@riojasalud.es (E.R.-E.); 2 Castilla-La Mancha Health Service, Illescas Primary Care Health Center, 45200 Toledo, Spain; 3 Department of Nursing, Universidad Católica de Ávila, 05005 Ávila, Spain; 4 Health Research Institute of Aragon (IIS), Group B21-20R, 50009 Zaragoza, Spain; bolivan@unizar.es (B.O.-B.); med000764@gmail.com (R.M.-B.); masanchezc@salud.aragon.es (M.A.S.-C.); 5 Aragonese Primary Care Research Group, redIAPP Group 016/07/01, 50009 Zaragoza, Spain; 6 Emergency Department, Miguel Servet University Hospital, 50009 Zaragoza, Spain; 7 University of Zaragoza, 50009 Zaragoza, Spain; 8 Aragon Health Service, Arrabal Primary Care Health Center, 50009 Zaragoza, Spain; 9 Department of Sociology, Social Work and Public Health, Faculty of Labour Sciences, University of Huelva, 21007 Huelva, Spain; 10 Safety and Health Posgraduate Program, Universidad Espíritu Santo, 092301 Guayaquil, Ecuador; 11 Aragon Health Service, Fuentes Norte Primary Care Health Center, 50002 Zaragoza, Spain; 12 La Rioja Health Service, San Pedro Hospital, 26006 Logroño, Spain; 13 La Rioja Health Service, Puerta de Arnedo Primary Care Health Center, 26580 Arnedo, Spain

**Keywords:** responsiveness, health services, primary care, patients expectations, human rights

## Abstract

A health system’s responsiveness is the result of patient expectations for the non-medical care they receive. The objective of this study was to assess mental patients’ responsiveness to the health system in primary care, as related to the domains of dignity, autonomy, confidentiality, and communication. Data were collected from 215 people over the age of 18 with mental disorders, using the Multi-Country Survey Study (MCSS) developed by the World Health Organization. Of them, 95% reported a good experience regarding the dignity, confidentiality, communication, and autonomy domains. Regarding responsiveness, patients valued the dignity domain as the most important one (25.1%). Among the patients who experienced poor confidentiality, five out of seven earned less than 900 euros per month (Χ^2^ = 10.8, *p* = 0.004). Among those who experienced good autonomy, 85 out of 156 belonged to the working social class (90.4%), and among those who valued it as poor (16.1%), the highest proportion was for middle class people (Χ^2^ = 13.1, *p* = 0.028). The two students and 87.5% of retirees experienced this dimension as good, and most patients who valued it as poor were unemployed (43.5%) (Χ^2^ = 13.0, *p* = 0.011). Patients with a household income higher than 900 euros more frequently valued responsiveness as good, regarding those domains related to communication, with OR = 3.84, 95% CI = 1.05–14.09, and confidentiality, with OR = 10.48, 95% CI = 1.94–56.59. To conclude, as regards responsiveness in primary care, the dignity domain always obtained the best scores by people with mental disorders. Low economic income is related to a poor assessment of confidentiality. Working class patients, students, and retirees value autonomy as good.

## 1. Introduction

Primary health care has been considered to be a fundamental part of the health system since the Declaration of Alma-Ata, for it makes essential care available to all individuals and the community, based on effective methods and technologies, and at an affordable cost for the society [1]. For its part, the Declaration of Astana advocates strengthening primary care with quality, safe, comprehensive, and integrated services [2] to improve the physical and mental health of people.

According to Ritchie and Roser, it is estimated that 792 million people lived with a mental health disorder by 2017. This is slightly more than one in ten people globally (10.7%), anxiety and depression disorders being the most common [3]. The prevalence of psychological distress in Spain was 19.1% in 2017, being higher in women (22.8%) than men (14.6%) [4].

Given the high prevalence of mental disorders and the increased costs of their care, it is necessary to promote primary care services as first-level care, for these are efficient and accessible [5]. Since access to primary care by mental health patients varies by sex and sociodemographic factors such as place of residence and socio-economic or cultural level, these variables should be considered to determine the distribution of geographical resources [6].

Another aspect to consider is the quality of care, which can refer to both clinical and non-clinical care [7]. Considering the latter, the WHO has developed the concept of health system responsiveness, defined as “the ability of the health system to meet the population’s legitimate expectations regarding their interaction with the health system, apart from expectations for improvements in health or wealth”; that is, it includes how well the health system meets the legitimate expectations of the population for the non-health-enhancing aspects of the health system. Eight domains are established, some of which are related to the rights of patients as human beings: dignity, autonomy, confidentiality, and communication, while others are structural (prompt attention, basic quality of facilities, access to social support networks, and choice of care provider) [8].

Dignity relates to the value of people as such. Treating people with dignity is tantamount to respecting them as valuable individuals [9]. In the context of health or illness, dignity also has a subjective dimension related to the person’s experience with his or her pathological process and the associated behaviour of health professionals [10]. A systematic review of the experience of patients admitted to mental health units confirmed the importance of accounting for their perspectives, their previous experiences, and preferences, which are key components in patient-centred care [11].

Although paternalism has a long tradition in psychiatry, in recent decades, the development of autonomy has come to displace it. However, there are still some issues that generate controversy among health professionals, such as the fact that these patients often have their autonomy diminished, which could imply serious consequences for them [12]. The Madrid Declaration on People with Mental Disorders emphasises the importance of reciprocity, understood as trust and mutual respect, to allow the patient to make free and informed decisions [13]. This involvement of patients in decision-making about their treatment, as well as active listening, is key to reducing the use of coercion in those with severe behavioural disorders [14].

The actions to carry out from the first level of care are related to the promotion of patient-centred care, recognising the reality of the problems that people with mental disorders face, talking to them without stigmatising them, and listening with empathy [15].

The development of information technologies has posed new challenges to confidentiality and privacy. Despite efforts to develop regulations and legislation to protect medical information, there is still no guarantee that this will be done to meet privacy standards at all levels [16].

Communication with people with mental health problems may be compromised by differences in culture, language, and religion, the use of coercive measures, a lack of understanding of overly technical language, or inadequate attitudes from professionals who end up ignoring or abusing patients [11,17]. Despite their scope, these issues may be minimised through the participation of professionals to make communication clearer and more intelligible. The practice of making shared decisions has been proposed as a strategy to promote communication between professionals and these patients [12]. Moreover, the implementation of shared decision-making has been associated with a reduction of fear and depression for the patient, and the improvement of their quality of life and satisfaction [18,19].

Responsiveness may be influenced by factors dependent on both the health system and the individual, such as their economic and cultural level, which are relevant for people with mental illnesses [20].

The WHO, in its Mental Health Action Plan 2013–2020, stated that health systems have not yet provided an adequate response to the burden of mental disorders and, consequently, the divergence between the need for treatment and its delivery is large worldwide [5].

The opinions of health service users and their perceptions of the quality of care are essential for changes and effective measures to be developed for the improvement of the service. New health policies based on social participation are needed to create services according to the needs of patients, which must include decent treatment.

These criteria should be considered by managers so as to adapt the responsiveness of health services to people’s expectations [21].

The objective of this study was to assess mental patients’ responsiveness to the health system in primary care, as related to the domains of dignity, autonomy, confidentiality, and communication.

## 2. Materials and Methods

This research work was conducted as part of the “Assessment of responsiveness of the primary care by the patients with mental illness and chronic disease” project, which assesses mental patients’ evaluations of primary care and compares them with the ones made by chronic patients.

A multicentre cross-sectional descriptive study was carried out at six health centres in the autonomous communities of Aragon and La Rioja (Spain) between January 2018 and June 2019.

Consecutive sampling was performed among patients who met the following inclusion criteria: being 18 years of age or older, having at least one mental disorder according to the ICD-10 (International Classification of Diseases, Tenth Edition) diagnostic criteria, being under pharmacological or psychological treatment at the time of inclusion in the study, having attended primary care consultations for any reason in the 12 months prior to the start of the study, not having a cognitive impairment, and not being under palliative care.

A short questionnaire, the Multi-Country Survey Study on Health and Responsiveness (MCSS), has been developed and validated by the WHO in 60 countries [22]. In this study, a portion of this questionnaire was used as a data collection instrument. This questionnaire is formed by seven defined domains for out-patient service users that may be divided into two categories: respect for persons and client orientation.

As a result, the responsiveness of the health system in terms of respect for persons was considered, including the dimensions of autonomy, dignity, confidentiality, and communication (Table 1). To this end, the participants were surveyed on their experiences of contact with the health system in the last 12 months.

A Likert scale with five sorted categories from 1 (very good) to 5 (very poor) is used here. To measure the importance of domains, participants were asked which domain was most important to them.

Secondary variables included all factors that could influence the assessment of the health system’s responsiveness, such as age, sex, level of education, occupation, subjective social class, and household income (monthly salaries of all household members). To determine social class, the participants were asked to choose among working, middle, or high class.

Sources for data collection were electronic medical history and face-to-face interviews with patients, which were conducted by trained external surveyors of the health care system.

Data analysis is described in Table 2.

The SPSS software version 24 (IBM: Armonk, NY, USA) was used for analysis.

### Ethics Approval

The authors assert that all procedures contributing to this work complied with the ethical standards of the Helsinki Declaration of 1975, as revised in 2008.

All of the subjects completed a written informed consent form, and their data were anonymised.

The informed consent was approved, together with the research protocol, by the Research Ethics Committee of Aragon, which is made up of collegiate professionals from different disciplines (C.I. PI 17/194), and by the Ministry of Science, Innovation and Universities of Spain, by the Regional Development European Funds (FEDER).

The subjects of study did not have cognitive impairments, so the written informed consent was obtained directly from them.

## 3. Results

The selected group consisted of 266 patients, of whom 36 refused to participate and 15 were excluded for not meeting the inclusion criteria. Finally, 215 people with mental disorders who had had at least one primary care consultation in the previous 12 months were interviewed. The characteristics of the sample are presented in Table 3. Of the participants, 60.5% believed that the main goal of a health service should be health care and, secondly, improving the people’s treatment when they receive medical care (31.2%).

When asked about the treatment received, 86.6% of patients stated that healthcare professionals have always treated them with respect, and for 95.3% of them, physical examinations were always carried out while preserving privacy. As for active listening, 82.8% claimed that health professionals always listened to them carefully, and 85.1% stated they have always explained things to them in an understandable way. In 50.7% of patients, physicians and nurses always let them participate in treatment or testing decisions.

Regarding responsiveness, patients valued the dignity domain as the most important one (25.1%), followed by communication (16.7%), confidentiality (6%), and autonomy (0.5%).

The patients’ experiences had been good in 95.8% of cases regarding dignity, 95.3% for confidentiality, 93.5% for communication, and 84% for autonomy.

There was an association between confidentiality and economic income. Among those who experienced good responsiveness regarding the confidentiality dimension, the largest percentage had an income of 901–1350 euros per month (98.6%). In addition, most patients who experienced it as poor earned less than 900 euros per month (13.3%) (*X*^2^ = 10.8, *p* = 0.004).

The autonomy dimension also appeared to be related to social class and employment situation. Most of those who experienced good autonomy belonged to the working social class (90.4%), and among those who valued it as poor (16.1%), the highest proportion was for middle-class people (*Χ*^2^ = 13.1, *p* = 0.028). A total of 87.5% of retirees experienced this dimension as good, and most patients who valued it as poor were unemployed (43.5%) (*Χ*^2^ = 13.0, *p* = 0.011) (Table 4).

The responsiveness for the communication domain was valued as poor by 6.5% of patients, the largest percentage corresponding to those with primary and secondary studies (*Χ*^2^ = 1.3, *p* = 0.503). There were no significant differences between the four responsiveness domains and sex, age, or marital status.

Logistic regression showed a significant outcome in the autonomy domain for the working social class, with OR = 0.36, 95% CI = 0.15–0.83, and for the unemployed, with OR = 3.33, 95% CI = 1.07–10.42.

Patients with household incomes higher than 900 euros more frequently valued responsiveness as good regarding those domains related to communication, with OR = 3.84, 95% CI = 1.05–14.09, and confidentiality, with OR = 10.48, 95% CI = 1.94–56.59.

When adjusting by social class, having a household income higher than 900 euros increased the probability of assessing confidentiality as good (*p* = 0.003), with OR = 14.1, 95% CI = 2.4–80.7.

## 4. Discussion

In this study, we considered mental disorder patients’ assessments of primary care responsiveness regarding domains related to respect for persons.

The results suggest that a high proportion of participants valued their experience with dignity, confidentiality, and communication as good, this percentage being somewhat lower in relation to autonomy. We also found that some sociodemographic characteristics, such as economic income, social class, and employment situations of people with mental disorders, could determine their experience with primary care. Autonomy was the most poorly assessed domain, as happened in other studies [23,24,25].

In the study by Forouzan et al. [23], conducted in Iran’s mental health system, the authors found that the best-valued responsiveness domains were confidentiality (92.4%) and dignity (81.8%), but only 42.7% of patients valued autonomy as good. In our work, these three dimensions also appeared as the best rated, but quantitatively there was a higher proportion of people with mental disorders who considered them good. In another study on out-patients who were receiving a follow-up by mental health services, autonomy was the most important domain, but it got a worse score regarding responsiveness. However, dignity and communication were the best rated [24].

The wish to take part in decision-making is frequent among mental health patients. Thus, their expectations about their autonomy are high [26]. When the participation of these patients is scarce, the autonomy dimension is more poorly valued. It is possible that, among primary care physicians, there is still a paternalistic attitude towards people with mental disorders, which may explain the poor assessment of responsiveness regarding this domain [27].

Confidentiality was rated as poor by patients who earned less than 900 euros per month. These results were consistent with those found in the Bramesfeld et al. [24] study, where responsiveness was worse in lower-income patients. In the work by Tille et al. [9], carried out on patients with different pathologies who attended general practitioner consultations, confidentiality was associated with the employment situation, being valued as poor by those who had a part-time job.

In previous studies, low income has been related to poor assessment of responsiveness in all the domains [28].

In terms of communication, we found poor responsiveness in patients with primary and secondary studies, although the differences were not significant. These findings are consistent with those of Tille et al. [9], which described this relationship considering patients with intermediate studies.

In another study, responsiveness regarding the communication domain was assessed as poor by people with higher economic incomes [29].

The disparities found in responsiveness regarding the socio-economic level and the level of education may be due to the fact that physicians tend to communicate less often with patients with lower economic incomes, as they believe they may be less involved in decision-making and also have difficulties understanding the information [30]. However, patients expect their physicians to provide them with information and carefully listen to them [31].

The differences found with our study may be due to the fact that, in our study, responsiveness had been assessed regarding primary care, where people with mental disorders can be assisted due to their illness or any other medical process, unlike in mental health services.

### Limitations

The main limitation of this study is the selection bias due to consecutive sampling, which was performed by selecting the sample from patients who attended primary care appointments and met the inclusion criteria. The potential for selection bias arising from voluntary participation is also acknowledged. Another limitation is that only four domains were considered among the ones identified by the WHO, as these were the four domains related to respect for persons.

Information bias was minimised by conducting interviews with trained, non-health care related interviewers, as well as by using an ad hoc designed data collection notebook for the study.

## 5. Conclusions

This study presents the experiences of people with mental disorders in relation to the non-medical care they receive in primary care. In particular, the responsiveness domains concerning respect for persons have been assessed.

The most important domain considered was dignity. Most participants considered their experience in relation to dignity, confidentiality, and communication as good, and, in a lower percentage, they rated autonomy as good as well.

Low economic income was related to a poor assessment of confidentiality. Working-class patients and retirees valued autonomy as good.

These results should serve to prioritise the implementation of measures that have the aim of improving the quality of primary care services provided to people with mental disorders. In this sense, from the perspective of non-medical care, communication with patients should be reinforced, offering complete and objective information so that users can make an autonomous decision about their healthcare. Health professionals must adapt communication to the needs of mental health patients, adjusting language to favour interaction and check understanding.

Data confidentiality must be guaranteed, not only regarding information about their health status, but also their personal circumstances.

To promote mental health patients’ dignity in health services, discrimination and stigmatisation must be avoided, favouring user participation in decision-making and respecting their autonomy.

## Figures and Tables

**Table 1:** Responsiveness domains regarding respect for persons.

Domain	Definition
Dignity	Being treated with respect by health professionals. Keeping privacy during physical examinations and treatment.
Confidentiality	Performing tests in a way that privacy is preserved. Keeping confidentiality of data offered by the patient and those regarding his or her illness.
Autonomy	Involving the patient, if wanted, in decision-making about his or her care or treatment, having the opportunity to reject it given no mental impairment. Asking for permission prior to performing tests or prescribing a treatment.
Clear communication	Offering the patients information about their health situation in a comprehensible way. Creating an intimate dialogue between the patient and the health professional. Listening carefully. Providing enough time for patients and relatives to ask any questions.

**Table 2:** Statistical analysis.

‘Responsiveness’ Variable	Descriptive Analysis	Bivariate Analysis	Multivariate Analysis
Dignity	Dichotomising: Good responsiveness with “good” and “very good” responses.	Contingency tables: To compare the sociodemographic factors with responsiveness.	Logistic regression: To assess the effect of the household income, social class, occupation, and level of education variables on each of the responsiveness domains.
Confidentiality	Poor responsiveness with “normal”, “bad”, and “very bad” as responses.	Chi-squared test or Fisher’s exact test for 2 × 2 tables with small counts.	
Autonomy	Absolute and relative frequencies.		
Clear communication	Means and standard deviations.	*p* < 0.05 was taken as significant.	95% CIs were calculated.

**Table 3:** Population sociodemographic characteristics.

Population Characteristics		
Age, *n* $\bar{x}$ (SD)	Variables	215, 62 (17.3)
Sex *n* (%)	Women	165 (76.7)
	Men	50 (23.3)
Marital status *n* (%)	Single	28 (13.0)
	Separate	23 (10.7)
	Married or with a partner	122 (56.7)
	Widow or widower	42 (19.5)
Social class *n* (%)	Working	99 (46.0)
	Middle	100 (46.5)
	High	3 (1.4)
Level of studies *n* (%)	Primary	111 (51.6)
	Secondary	86 (40.0)
	University	18 (8.4)
Income *n* (%)	<900 €	38 (17.7)
	901–1350 €	70 (32.6)
	>1350 €	62 (28.8)
Employment situation *n* (%)	Employee	41 (21.9)
	Student	2 (1.1)
	Unemployed	23 (12.3)
	Retiree	116 (62.0)
	Self-employed	5 (2.7)

SD = standard deviation; $\bar{x}$: mean.

**Table 4:** Association between responsiveness and patients’ economic income, social class, and employment situations.

Responsiveness		Good *n* (%)	Poor *n* (%)	*p*
**Confidentiality**	**Income**			0.004 *
	<900 €	31 (86.1)	5 (13.9)	
	901–1350 €	69 (98.6)	1 (1.4)	
	>1350 €	61 (98.4)	1 (1.6)	
**Autonomy**	**Social Class**			0.028 *
	Working	85 (90.4)	9 (9.6)	
	Middle	70 (77.8)	20 (22.5)	
	High	1 (50.0)	1 (50.0)	
	**Employment Situation**			0.011 *
	Employee	31 (83.8)	6 (16.2)	
	Student	2 (100)	0 (0)	
	Unemployed	13 (56.5)	10 (43.5)	
	Retiree	91 (87.5)	13 (12.5)	
	Self-employed	4 (80.0)	1 (20.0)	

* Chi-squared and Fisher’s exact test statistically significant at <0.05.
